# Dose outside of the prostate is associated with improved outcomes for high-risk prostate cancer patients treated with brachytherapy boost

**DOI:** 10.3389/fonc.2023.1200676

**Published:** 2023-06-15

**Authors:** Jane Shortall, Eliana Vasquez Osorio, Andrew Green, Alan McWilliam, Thriaviyam Elumalai, Kimberley Reeves, Corinne Johnson-Hart, William Beasley, Peter Hoskin, Ananya Choudhury, Marcel van Herk

**Affiliations:** ^1^ Division of Cancer Sciences, Faculty of Biology, Medicine and Health, The University of Manchester, Manchester, United Kingdom; ^2^ Department of Radiotherapy Related Research, The Christie National Health Service (NHS) Foundation Trust, Manchester, United Kingdom

**Keywords:** radiation oncology, image based data mining, prostate, outcomes, radiotherapy

## Abstract

**Background:**

One in three high-risk prostate cancer patients treated with radiotherapy recur. Detection of lymph node metastasis and microscopic disease spread using conventional imaging is poor, and many patients are under-treated due to suboptimal seminal vesicle or lymph node irradiation. We use Image Based Data Mining (IBDM) to investigate association between dose distributions, and prognostic variables and biochemical recurrence (BCR) in prostate cancer patients treated with radiotherapy. We further test whether including dose information in risk-stratification models improves performance.

**Method:**

Planning CTs, dose distributions and clinical information were collected for 612 high-risk prostate cancer patients treated with conformal hypo-fractionated radiotherapy, intensity modulated radiotherapy (IMRT), or IMRT plus a single fraction high dose rate (HDR) brachytherapy boost. Dose distributions (including HDR boost) of all studied patients were mapped to a reference anatomy using the prostate delineations. Regions where dose distributions significantly differed between patients that did and did-not experience BCR were assessed voxel-wise using 1) a binary endpoint of BCR at four-years (dose only) and 2) Cox-IBDM (dose and prognostic variables). Regions where dose was associated with outcome were identified. Cox proportional-hazard models with and without region dose information were produced and the Akaike Information Criterion (AIC) was used to assess model performance.

**Results:**

No significant regions were observed for patients treated with hypo-fractionated radiotherapy or IMRT. Regions outside the target where higher dose was associated with lower BCR were observed for patients treated with brachytherapy boost. Cox-IBDM revealed that dose response was influenced by age and T-stage. A region at the seminal vesicle tips was identified in binary- and Cox-IBDM. Including the mean dose in this region in a risk-stratification model (hazard ratio=0.84, p=0.005) significantly reduced AIC values (p=0.019), indicating superior performance, compared with prognostic variables only. The region dose was lower in the brachytherapy boost patients compared with the external beam cohorts supporting the occurrence of marginal misses.

**Conclusion:**

Association was identified between BCR and dose outside of the target region in high-risk prostate cancer patients treated with IMRT plus brachytherapy boost. We show, for the first-time, that the importance of irradiating this region is linked to prognostic variables.

## 1 Introduction

Radiotherapy is the primary curative treatment for approximately 30% of prostate cancer patients in the UK, and has been attributed to high overall survival rates (1). However, prognosis remains poor for high-risk patients (2, 3), and 20-30% of patients experience biochemical recurrence (BCR) within five years of radiotherapy (4).

There is currently no consensus for the optimal management of high-risk prostate cancer (2), and current standard of care radiotherapy is delivered to the prostate and seminal vesicles only, as opposed to lymph node and Whole Pelvis Radiotherapy (WPRT) which has shown some benefit for some high-risk patients (2, 5–10). Some patients may receive lymph node radiotherapy, however this is administered using risk-stratification models that consider known prognostic factors, but have limited consideration for the risk of microscopic spread of disease (2, 11–15).

Advances in diagnostic imaging, such as prostate-specific membrane antigen positron emission tomography (PSMA-PET) imaging has revealed that microscopic disease spread to regions outside of the usual pelvic lymph nodes are more common than previously thought (16, 17). With radiotherapy dose distributions becoming increasingly more conformal around the prostate, many patients are potentially left with under- or un-treated lymph node or microscopic disease outside of the target volume, which could consequently lead to BCR (18–22).

Clinical studies and trials comparing lymph node and WPRT with prostate only radiotherapy (PORT) have conflicting outcomes (2, 5–10). Controversy associated with WPRT is likely due to inability to detect those at risk and to adequately treat involved lymph nodes or regions of microscopic disease whilst remaining within dose tolerances. As a result, WPRT often results in large volumes being irradiated with low doses, which is sub-optimal for both disease control and normal tissue complications.

Conversely, whilst radiotherapy techniques try to limit dose outside of the Planning Target Volume (PTV) to spare normal tissues, studies have shown that incidental dose outside of the PTV could be inadvertently treating subclinical disease (23). Witte et al. (18) and Chen et al. (24) used Image Based Data Mining technique (IBDM), where the local association between dose and outcome is explored on a per-voxel basis, and found that that incidental dose to the obturator was associated with improved outcomes (25), a region that had previously been associated with clinically relevant incidental dose (26). More recently, Witte et al. suggested that the relationship between incidental dose and BCR is influenced by fractionation when they identified a region in the in obturator internus muscles where incidental dose was beneficial for patients treated with standard fractionation radiotherapy, but not in patients treated with a hypo-fractionated schedule (27).

These IBDM studies just consider the dose distribution, and do not include known prognostic factors that current stratification models use. Green et al. presented a Cox-IBDM methodology, whereby a Cox regression is performed in every voxel of the dose distribution, allowing dose-sensitive regions to be identified considering both dose and prognostic clinical variables simultaneously (28). Our Cox methodology works per voxel using permutation testing to correct for multiple testing.

We, firstly, aim to explore the hypothesis that targeted dose outside of the prostate reduces BCR in a selection of high-risk prostate cancer patients (18). Secondly, we aim to investigate whether fractionation schedule influences this “extra-prostatic dose-effect relation” (27). Thirdly, we apply Cox-IBDM, accounting for both dose and prognostic variables to develop a predictive model to select high-risk prostate cancer patients that may benefit from adapted target volumes.

## 2 Method

We refer the reader to **Supplementary Material Appendix A** for more detailed methodology.

### 2.1 Study design

A total of 612 patients with high-risk prostate cancer (2) treated with radiotherapy between 2005 and 2013 at a single academic center were included. Institutional approval had been granted to use this data (research ethics committee reference: 17/NW/0060). Patients were treated with either conformal hypo-fractionated radiotherapy (50Gy in 16 fractions (equivalent dose in 2Gy fractions (EQD2)=66.07Gy, using alpha-beta ratio=1.5), n=258, prostate and 1-2cm proximal seminal vesicles irradiated, 2005-2011), hypo-fractionated Intensity Modulated Radiotherapy (IMRT) (57Gy or 60Gy in 19 or 20 fractions respectively (EQD2 = 73.29/77.14Gy), n=245, prostate and 1-2cm proximal seminal vesicles irradiated in line with CHHiP guidelines (29), 2008-2013), or IMRT (37.5 Gy to the prostate in 15 fractions) plus a 15Gy single fraction High Dose Rate (HDR) brachytherapy boost to the prostate and 1-2cm proximal seminal vesicles (n=109, (EQD2 = 113.57Gy), 2009-2013). Treatment fractionation schedule was assigned by consultant clinical oncologists according to local practice. No patients received elective nodal irradiation, and Androgen Deprivation Therapy (ADT) was the only systemic therapy used.

Planning Computed Tomography (CT) scan and delineations, 3D planned dose distribution (Philips Pinnacle treatment planning system archive) and patient and tumor characteristics (age, T-stage, Gleason grade, Androgen Deprivation Therapy (ADT) duration and baseline PSA) were collected for all patients. Note that no other systemic therapies than ADT were used

Brachytherapy boost was planned using ultrasound imaging and a single PTV to the prostate and delivered as an HDR treatment using an Ir-192 source. As dose distribution export is not supported by the used brachytherapy planning system, the planned brachytherapy dwell positions and catheter positions and times were collected and used to reconstruct the planned dose using the dose distribution of a nominal 10Ci Ir-192 source (30) and the recorded dwell position in the DICOM plan object (31). The dose distributions of each dwell position were then summed, accounting for contributions from all sources, to provide the total dose. See **Supplementary Material Appendix A** section 1.1 for more details on brachytherapy dose reconstruction.

The end-point of the study was BCR (PSA nadir + 2ng/ml) (32), and the three cohorts were analyzed separately.

### 2.2 Dose mapping

For brachytherapy boost, the reconstructed dose distribution was spatially aligned to the external beam planned dose distribution and the total treatment dose summed using EQD2 dose (33) (**Supplementary Material Appendix A** section 2). Note that, as analysis explored relative differences between dose distributions of the same treatment technique and not absolute dose, the alpha-beta ratio is not critical in the range of relevant alpha-betas (34). Dose reconstruction and alignment was visually checked for 10 arbitrary patients to ensure the 15Gy line aligned with the prostate contour. Sensitivity for uncertainties in the dose summation were simulated by randomly shifting the brachytherapy dose by up to 1cm in any cardinal direction (as to simulate a worst-case scenario) and repeating analysis.

Next, planning CTs were spatially registered, and dose distributions mapped to an arbitrarily chosen reference patient. A region of interest forming a sphere of approximately 10cm radius, centered on the centre of the reference prostate, was chosen for analysis to remove regions where spurious results could occur due to differences in radiotherapy plans depending on patient anatomy. See **Supplementary Material Appendix A** section 2 for details of algorithm used.

Prior to dose mapping, dose distributions of all patients were flipped in the left-right direction and included in analysis twice. This method, which has been commonly used in other IBDM studies (35), assumes that the likelihood of microscopic disease is symmetric, avoids spurious laterality biased results caused by small asymmetries in the dose distributions, and improves statistical power.

To assess and account for the accuracy of the dose mapping, Target Registration Error analysis was performed (36) and dose distributions blurred using a 3D Gaussian filter of width according to the standard deviation in each cardinal direction of manually made landmarks on the seminal vesicle tips and apex for a selection of patients (LR: 0.49, AP: 0.65, SI: 0.91cm, **Supplementary Material Figure S1**).

### 2.3 Voxel-based analysis

Binary-IBDM and Cox-IBDM were performed to assess differences in the dose distributions of patients who did and did-not recur (in-house software (37)). For this, mapped doses were grouped depending on BCR status (PSA failure free survival (bNED)=0, fail=1).

Binary-IBDM: Mapped doses for each voxel of the bNED and fail at four-years post radiotherapy (18) groups were compared using a Students T-test. T-maps containing the observed t-values in each voxel were created. A positive t-value indicates that excess dose is associated with reduced BCR.

Cox-IBDM: For Cox-IBDM, a Cox proportional-hazards model was constructed for each voxel, including mapped doses to that voxel, age (continuous), T-stage (≥T3 vs <T3), Gleason grade (≥8 vs <8), ADT duration (≥18 months vs <18months), and baseline PSA (continuous). Categorical variables T-stage and Gleason grade were dichotomized based on the distribution of patient demographics (by their median). ADT was dichotomized at 18 months in accordance with the National Comprehensive Cancer Network guidelines, which recommend a significant benefit to prescribing ADT for over 18 months to high-risk patients (38, 39). Similar to binary-IBDM, resulting β-statistics were collected to create β-maps. To ease interpretability, the observed β-maps and was transformed to Hazard Ratio (HR) maps, by applying the exponential. Patients with missing prognostic variables were excluded. **Figure S2** in **Supplementary Material** summarizes patients included in each stage of the analysis.

To correct for multiple testing (24, 37, 40), and to determine regions of significance, permutation testing (n=1000) was performed (37, 40, 41). Event labels were randomly permuted, and a distribution of extreme statistics (T or beta) were derived, keeping the null hypothesis of no difference between BCR events. A threshold of corresponding to p =0.02, corresponding to 98-th percentile in this extreme statistics distribution, was used to identify significant regions. Iso-t- and iso-beta-levels indicating the significant regions indicate regions where the dose distributions of patients who did and did not recur significantly differed (37, 41). For Cox-IBDM, iso-β levels indicating significance were plotted on the observed β-map for each variable (see Green et al. for more detail (37)).

We refer the reader to **Supplementary Material Appendix A** section 4.1 and 4.2 for more details on our Binary- and Cox-IBDM methods, and to (37, 40–42) for more detailed explanation of the statistical methods used.

### 2.4 Statistical analysis

The mean dose of the regions commonly identified for both binary- and Cox-IBDM were collected. Univariable and multivariable Cox proportional-hazard models not including and including these mean region doses were produced and the Akaike information criterion (AIC) and concordance-index (c-index) of each model compared to assess model performance. All statistical analysis was performed using R (version 4.0.2) in RStudio (desktop version 1.3.1073).

## 3 Results

### 3.1 Baseline characteristics

Patient demographics are summarized in **Table 1**. There was no significant difference in recurrence between cohorts (p=0.49) (**Supplementary Material Figure S3**). There were significant differences in age, T-stage, ADT duration and Gleason grade (p ≤ 0.033) between the three cohorts and the distribution of most variables was significantly different between the brachytherapy patients and at least one other cohort (**Supplementary Material Figure S4**).

**Table 1:** Patient demographics for the 612 high-risk prostate cancer patients selected for this study.

	Conformal hypo-fractionated (50Gy in 16#)	IMRT (57/60Gy in 19/20#)	IMRT + Brachytherapy (37.5Gy in 15# + 15Gy HDR boost)
**Cohort size**	258	245	109
**Mean age (years)**	68	67	65
**Median follow-up (years)**	5.07	5.80	4.79
T-Stage			
** T1**	43	15	10
** T2**	65	67	47
** T3**	144	158	52
** T4**	3	5	0
** NA**	3	0	0
**Median T-Stage**	3	3	2
Gleason grade			
**6**	44	22	6
**7**	111	105	52
**8**	46	58	24
**9**	52	56	26
**10**	3	3	1
**NA**	2	1	0
**Median Gleason (range)**	7 (6-10)	7 (6-10)	7 (6-10)
**ADT (Yes/No/NA)**	242/3/13	191/0/23	109/0/0
**Median ADT duration (months)**	9	27	18
**ADT duration >18 months**	75	114	59
**Mean baseline PSA (ng/ml)**	40.04	32.05	26.67
**Median baseline PSA (ng/ml)**	24.00	21.00	22.00
**Number of BCR during follow-up**	124	77	33
**Number of 4-year BCR**	49	49	24
**Number of 5-year BCR**	69	60	27
**Mean recurrence time (years)**	5.57	5.11	4.55
**Median recurrence time (years)**	5.24	5	4.64

**Tables 2A, B** show univariable and multivariable analysis for prognostic variables. No variable was significant across all cohorts. No variables were significant for brachytherapy patients in univariable analysis, but T-stage was significant (p=0.048, HR: 0.46 (0.22, 0.99)) in multivariable analysis. For interest, we performed multivariable analysis dichotomizing Gleason class ≥3 vs ≤2, i.e. ≥4 + 3 vs ≤3 + 4, as according to The International Society of Urological Pathology (ISUP), as opposed to ≥8 vs <8, i.e. ISUP Class 4-5 vs ≤3 (43) (**Supplementary Material Table S1**). Results were not different for any cohort.

**Table 2:** Univariable (panel A) and multivariable (panel B) Cox proportional-hazards analysis for clinical prognostic covariates included in the study (age, T-stage, Gleason grade, ADT duration, baseline PSA).

A						
Univariable	Conformal hypo-fractionated (50Gy in 16#)		IMRT (57/60Gy in 19/20#)		EBRT + Brachytherapy (37.5Gy in 15# + 15Gy HDR boost)	
	HR (95% CI)	p-value	HR (95% CI)	p-value	HR (95% CI)	p-value
Age (continuous)	0.98 (0.95 - 1.00)	0.100	0.97 (0.94 - 1.01)	0.130	0.96 (0.91 - 1.01)	0.110
T-stage (≥T3 reference)	–	–	–	–	–	–
<T3	0.68 (0.47 - 0.98)	**0.040**	0.70 (0.42 - 1.15)	0.200	0.58 (0.28 - 1.19)	0.140
Gleason grade (≥8 as reference)	–	–	–	–	–	–
<8	0.7 (0.49 - 1.00)	0.050	0.79 (0.50 - 1.25)	0.300	0.78 (0.38 - 1.57)	0.500
ADT duration group (≥18 months as reference)	–	–	–	–	–	–
<18 months	0.93 (0.64 - 1.36)	0.700	1.48 (0.87 - 2.52)	0.140	1.32 (0.65 - 2.68)	0.400
Baseline PSA (ng/ml)	1.00 (1.00 - 1.00)	**0.019**	1.00 (1.00 - 1.01)	**<0.001**	1.01 (0.99 - 1.02)	0.200

B						
Multivariable	Conformal hypo-fractionated (50Gy in 16#)		IMRT (57/60Gy in 19/20#)		EBRT + Brachytherapy (37.5Gy in 15# + 15Gy HDR boost)	
	HR (95% CI)	p-value	HR (95% CI)	p-value	HR (95% CI)	p-value
Age (continuous)	0.98 (0.95 - 1.01)	0.200	1.00 (0.95 - 1.04)	0.900	0.95 (0.90 - 1.00)	0.071
T-stage (≥T3 reference)	–	–	–	–	–	–
<T3	0.69 (0.47 - 1.02)	0.062	0.43 (0.22 - 0.83)	**0.012**	0.46 (0.22 - 0.99)	**0.048**
Gleason grade (≥8 as reference)	–	–	–	–	–	–
<8	0.72 (0.48 - 1.07)	0.110	0.60 (0.32 - 1.00)	0.100	0.59 (0.28 - 1.24)	0.200
ADT duration group (≥18 months as reference)	–	–	–	–	–	–
<18 months	1.19 (0.78 - 1.83)	0.400	1.86 (1.00 - 3.48)	0.051	2.12 (0.95 - 4.72)	0.067
Baseline PSA (ng/ml)	1.00 (1.00 - 1.00)	**0.015**	1.01 (1.00 - 1.01)	**<0.001**	1.01 (1.00 - 1.02)	0.200
HR = Hazard Ratio	CI = Confidence Interval				AIC = 264.4, c-index = 0.652	

Analysis was conducted on each fractionation cohort separately. Bold means Statistically significant.

### 3.2 Binary-IBDM

**Figure 1:**
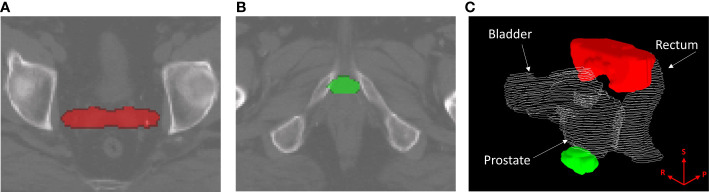
Regions where the dose distribution was significantly different (p ≤ 0.02) between patients who did and did-not experience biochemical recurrence (BCR) within four years of radiotherapy, for patients treated with intensity modulated radiotherapy plus a single high dose rate brachytherapy boost. Regions are overlayed on the planning CT of the reference patient used for image registration and dose mapping, and indicate where excess dose was associated with lower BCR. The identified region in **(A)**, marked in red, corresponds with the seminal vesicle tips. The region in **(B)**, marked in green, corresponds to the apex region of the prostate. **(C)** shows the two regions in relation to the prostate, rectum and bladder of the reference patient. The superior (S), right (R) and posterior (P) directions are indicated on the render in **(C)**.

Binary-IBDM did not identify any regions where the dose distribution was significantly different between recur status within four-years of radiotherapy for patients treated with hypo-fractionated radiotherapy or IMRT. However, two regions were identified for patients who were treated with brachytherapy boost: one at the seminal vesicle tips (red overlay, **Figures 1A**, **C**), and one at the apex of the prostate (green overlay, **Figures 1B**, **C**).

### 3.3 Cox-IBDM

**Figure 2:**
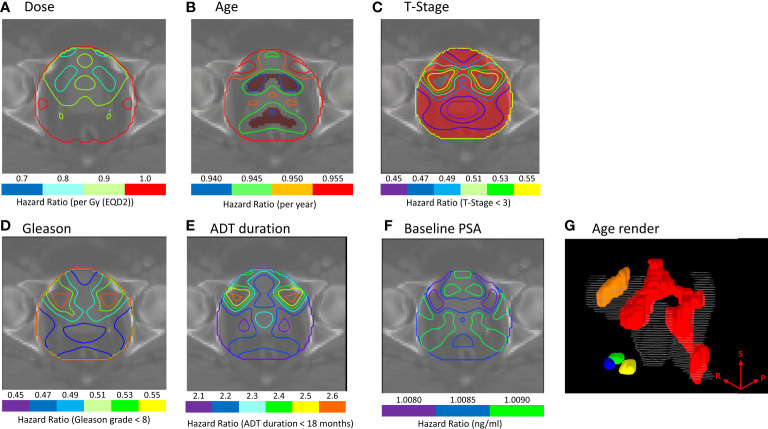
**(A–F)** Cox-IBDM results for patients treated with IMRT plus brachytherapy boost including all clinical variables and voxel-wise dose. Iso-Hazard ratios are indicated by the unfilled contours (note the different scales for each variable), and significance (p ≤ 0.02) is indicated by the filled red regions. **(G)** shows a 3D render of regions where age was significantly associated with biochemical recurrence (BCR); the seminal vesicles, extending down the posterior aspect of the rectum (red), the apex of the prostate (blue, green and yellow), and the superior aspect of the bladder (orange) were significantly associated with BCR. Results indicate that younger patients would benefit from excess dose in these regions. The superior (S), right (R) and posterior (P) directions are indicated on the render in **(G)**.

Similarly, there were no regions of significance for patients treated with hypo-fractionated radiotherapy or IMRT in our Cox-IBDM analysis (**Supplementary Material Figures S5, 6**). Association was identified for patients receiving a brachytherapy boost, indicated by the red regions in **Figures 2B, C** (p ≤ 0.02). Three regions were associated with age: one covering the seminal vesicles, extending from the tips down to the posterior aspect of the prostate and to the inferior posterior side of the rectum (red region **Figure 2G**), one superior to the bladder (orange region **Figure 2G**), and one close to the inferior aspect of the prostate (blue green and yellow regions **Figure 2G**). T-stage was significant in most of the anatomy (**Figure 2C**).

These maps indicate where clinical variables had a protective effect for a given dose (Age HR: 0.940-0.955, T-stage HR: 0.450-0.550). Analysis suggests younger patients, and patients with T-stage ≥T3, would benefit from additional dose in these regions to achieve the same BCR as older patients and patients with lower T-stage.

**Figure 3:**
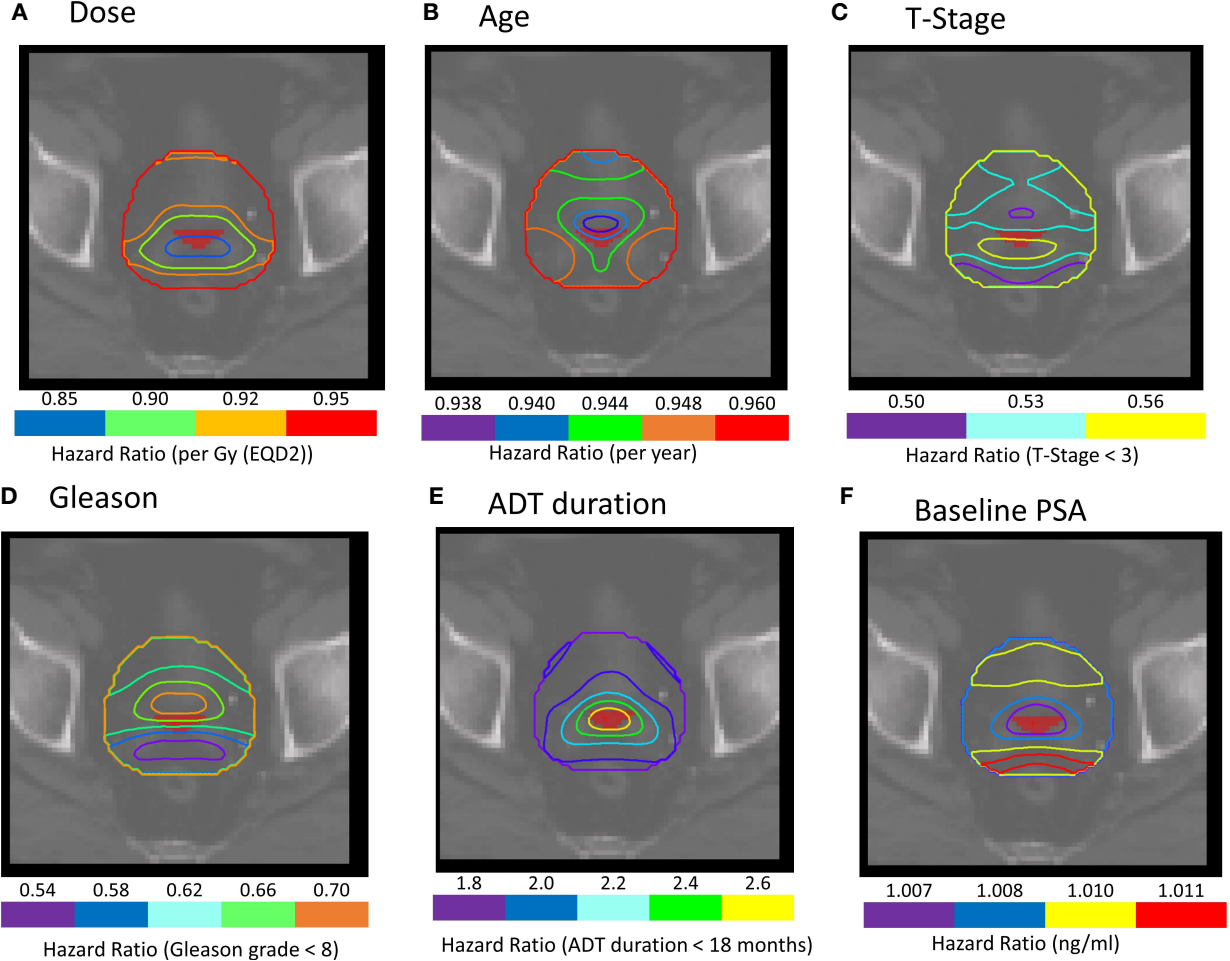
**(A–F)** Cox-IBDM results for patients treated with IMRT plus brachytherapy boost including all clinical variables and voxel-wise dose. Iso-Hazard ratios are indicated by the unfilled contours (note the different scales for each variable). The filled red region, close to the seminal vesicle tips, indicates a common region of significance identified in all IBDM analysis (p ≤ 0.02). Note that the filled red region represents the region in the dose distribution that was significant for binary-IBDM, and in Cox-IBDM when accounting for age and T-stage. **(B, C)** This region is indicated on all prognostic variables to exemplify the hazard ratio associated with each variable in this region.

**Figure 4:**
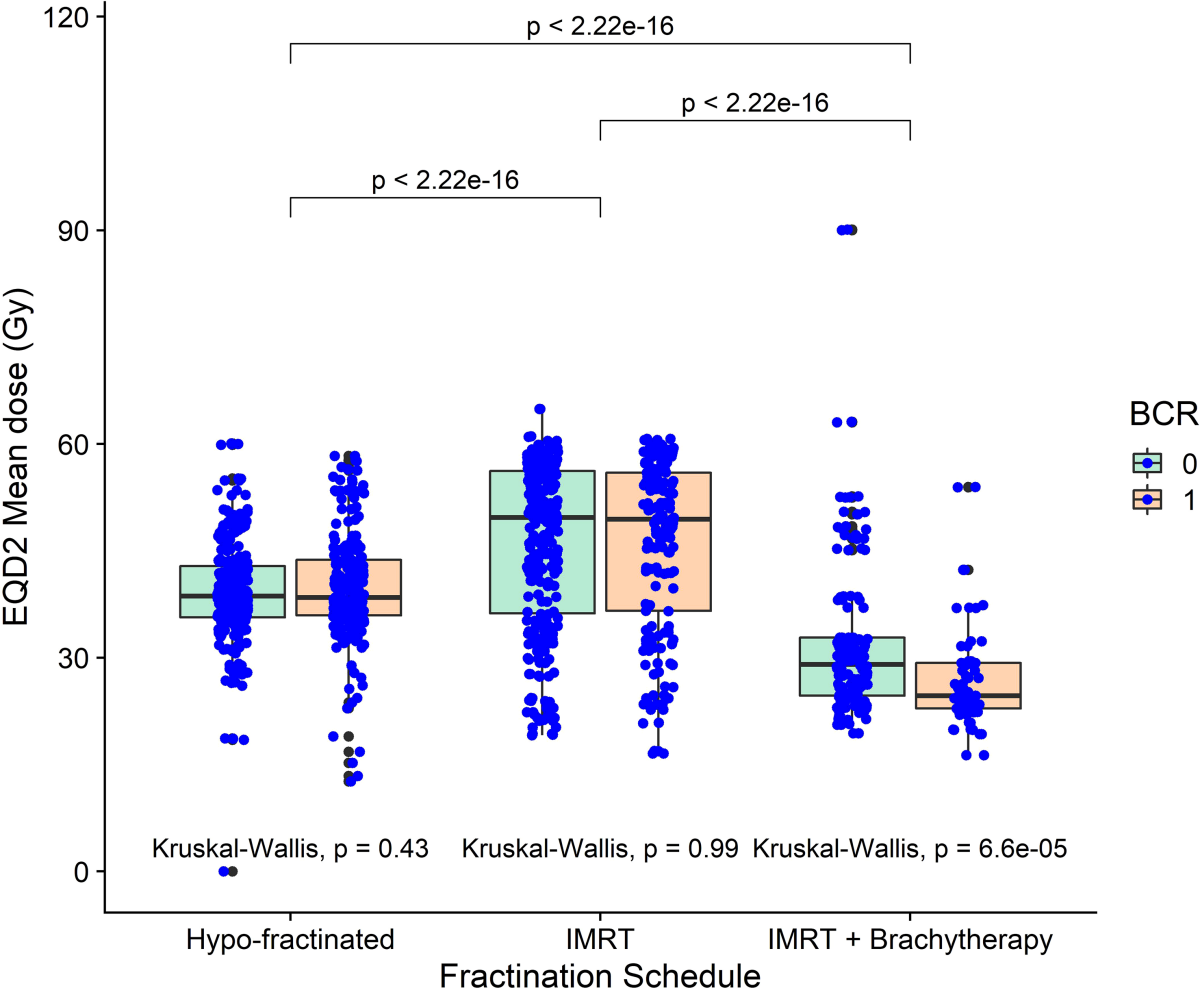
The mean dose in the region identified as a common region of significance for patients treated with externa, beam radiotherapy plus a single high dose rate brachytherapy boost, across all three cohorts. Mean dose was significantly lower in brachytherapy patients compared with the other two cohorts. Mean dose was also significantly lower for patients receiving brachytherapy that recurred, compared to those who didn’t.

The region indicated by red overlay in **Figures 3A–F**, close to the seminal vesicle tips in a different slice than presented in **Figure 2**, where binary-IBDM and Cox-IBDM results overlap. Planned dose in this region was significantly lower for all patients treated with brachytherapy compared with the other cohorts (**Figure 4**), and lower in brachytherapy patients that recurred, compared with brachytherapy patients who did not recur (p<0.0001). Interestingly, the planned dose was similar (p≥0.43) for patients that did and did not recur in the other two cohorts.

Univariable and multivariable analysis without and with dose information (**Figure 3**) are presented in **Table 3**. Dose in this region was significantly associated with BCR in univariable and multivariable analysis (HR:0.84-0.89, p ≤ 0.037). Results suggest higher dose in the distal seminal vesicles and higher age have a protective effect, and BCR was reduced by 16% per additional Gy (HR=0.84-0.94).

**Table 3:** Univariable and multivariable Cox proportional-hazards analysis without and with mean dose in the significant region common to all IBDM analysis (see **Figure 3**).

37.5 Gy in 15# + 15 Gy HDR Brachytherapy boost	Univariable		Multivariable clinical		Multivariable dose	
	HR (95% CI)	p-value	HR (95% CI)	p-value	HR (95% CI)	p-value
Dose overlap (binary- + Cox-IBDM)	0.89 (0.75 - 0.99)	**0.037**	–	–	0.84 (0.75 - 0.95)	**0.005**
Age (continuous)	0.96 (0.91 - 1.01)	0.110	0.95 (0.90 - 1.00)	0.071	0.94 (0.89 - 1.00)	**0.039**
T-stage (≥T3 reference)	–	–	–	–	–	–
<T3	0.58 (0.28 - 1.19)	0.140	0.46 (0.22 - 0.99)	**0.048**	0.56 (0.26 - 1.19)	0.130
Gleason grade (≥8 as reference)	–	–	–	–	–	–
<8	0.78 (0.38 - 1.57)	0.500	0.59 (0.28 - 1.24)	0.200	0.65 (0.31 - 1.36)	0.300
ADT duration group (≥18 months as reference)	–	–	–	–	–	–
<18 months	1.32 (0.65 - 2.68)	0.400	2.12 (0.95 - 4.72)	0.067	2.63 (1.14 - 6.05)	**0.023**
Baseline PSA (ng/ml)	1.01 (0.99 - 1.02)	0.200	1.01 (1.00 - 1.02)	0.200	1.01 (0.99 - 1.02)	0.300
HR = Hazard Ratio	**CI = Confidence Interval**		**AIC = 264.4, c-index = 0.652**		**AIC = 256.6, c-index = 0.722**	
			**p-value(ΔAIC) = 0.019**			

The AIC value is lower, and the c-index higher, in the model including dose information, indicating that model performance is improved when dose is considered. Bold means Statistically significant.

AIC significantly reduced from 264.4 to 256.6 (p=exp(-ΔAIC/2)=0.019), and c-index increased from 0.652 to 0.722, indicating improved model performance, when region dose was included.

## 4 Discussion

We have identified, using two independent voxel-based methods, regions outside of the target volume where planned dose relates to treatment outcome for high-risk prostate cancer patients treated with IMRT plus a single HDR brachytherapy boost. Excess dose in these regions was associated with lower BCR.

This is one of the first immerging implementations of a multivariable Cox-IBDM methodology for prostate cancer patients (40, 44). Not only do we show that the Cox-IBDM methodology reveals a spatial dependency of the association between prognostic variables and dose, but we show that performance of predictive models is improved when we consider dose.

Binary-IBDM identified a region at the distal seminal vesicles and apex of the prostate where excess dose was significantly associated with reduced BCR within four years of radiotherapy. Our results support previous observations that incidental dose outside the target region is associated with reduced BCR in high-risk prostate cancer patients (18, 24, 45), providing further evidence for adapting the treatment volume for men with high-risk disease. Further, the regions observed in both IBDM methods do cover part of the obturator region and external iliac nodes, supporting previous observations (18) and PSMA-PET studies (26) providing further evidence of nodal disease and for inclusion of nearby lymph nodes in the treatment volume for men with high-risk disease.

It is important to note that each study uses independent patient cohorts, treated with different schedules and protocols (18, 24). For example, in our patients, only the proximal 1-2cm of the seminal vesicles were included in the CTV, whereas in previous studies the whole of the seminal vesicles were included (18, 24). Our work suggests that seminal vesicle coverage is as important as lymph node irradiation, and external validation using cohorts with and without seminal vesicle coverage should be conducted to explore further.

Cox-IBDM identified similar regions where excess dose was associated with lower BCR. Results suggest that dose response was influenced by age and T-stage in brachytherapy patients. Regions proximal to the bladder, the seminal vesicles extending to the posterior of the meso-rectum, and the apex were significantly associated with BCR when adjusting for age (**Figure 2B**). Most of the region of interest was significantly associated with BCR when adjusting for T-stage (**Figure 2C**). Results suggest younger patients (HR: 0.935-0.955), and those with T-stage T3 and above (HR: 0.51-0.65), would benefit from more extensive coverage of the seminal vesicles.

Alike previous work, no significant association was observed for patients treated with hypo-fractionated radiotherapy (3.1Gy per fraction) or hypo-fractionated IMRT (3Gy per fraction) schedules (27). We note previous observations occur in patients treated with a conventional 2Gy per fraction schedule (27), not too dissimilar to the IMRT schedule of 2.5Gy per fraction the brachytherapy patients in our study. Our results support previous observations of an “extra-prostatic dose-effect relation” (27), and differences in dose response, that could occur around 2-2.5Gy per fraction, that we suggest depend on fractionation and differences in radiotherapy techniques. Although we do acknowledge that the 37.5Gy treatment course received by the brachytherapy patients is relatively low, confounded by the conformal dose distribution and operator dependency, the brachytherapy is too conformal to treat microscopic disease in higher-risk patients.

Despite the highest EQD2 prescription in the prostate, dose in the “common” region (**Figures 3**, **4**), which was outside of the CTV for 95 of the 109 brachytherapy patients, was significantly lower for brachytherapy patients compared with the other cohorts (**Supplementary Material Figures S7, 8**). As there was no difference in recurrence between the cohorts (**Supplementary Material Figure S3**), results indicate that the conformal brachytherapy dose could result in more failures at the distal seminal vesicles, potentially due to undetected and under-treated microscopic disease and seminal vesicle involvement, and less in the prostate. We further note that the majority of patients presented with Gleason grade ≥7 and high baseline PSA (≥10 ng/m) (46), and could have had undetected and under-treated micro-metastasis in the lymph nodes (18) with a delay in BCR due to hormone therapy. Our results strongly suggest that the CTV should be extended to include the entirety of the seminal vesicles in younger patients and those with T-stage ≥T3 (88/109 patients) treated with brachytherapy boost. However, it is appreciated that achieving full seminal vesicle coverage, which have large inter-fraction motion (47–49), would require margins of 8-10mm (48), and may therefore be difficult.

The conformal brachytherapy dose could also help explain the region identified near the meso-rectum (**Figure 2**). Although local recurrence is usually found in the prostate (50, 51), PSMA-PET has revealed that meso-rectal metastases occur in around 15% of prostate patients (16, 21, 22). It would have been interesting to explore whether prognostic variables or dose outside of the prostate influenced recurrence location, however we did not have access to this data.

Our results could indicate that microscopic seminal vesicle invasion was not detected at diagnosis, and some patients were consequentially under-staged and therefore under-treated. Patients included in this study were treated prior to 2013, and PSMA-PET was not available during diagnosis (CT or MRI and bone scan only). Our results highlight the importance of implementing advanced imaging techniques, we are currently evaluating the role of oxygen-enhanced MRI (OE-MRI) (52) or Restriction Spectrum Imaging (RSI) (53) in predicting the extent of microscopic disease spread.

Our Cox-IBDM results indicate that biological effects due to patient characteristics are likely. Brachytherapy patients were significantly younger than the other two cohorts (p ≤ 0.014) (**Supplementary Material Figure S4**). Evidence suggests that a patient’s biology is associated with the incidence and aggressiveness of disease (54–58), and that biology changes with age, causing more aggressive disease in younger patients (54, 58, 59). Results therefore suggest that some of the patients treated with brachytherapy had more aggressive cancer with microscopic disease that had spread outside of the conformal brachytherapy dose into the low dose regions identified in cox-IBDM and was consequently under-treated. Our results suggest that younger patients with aggressive disease may be less suitable for radiotherapy treatments with high levels of conformity such as HDR brachytherapy boost. This finding should, of course, be externally validated in other cohorts of conformal radiotherapy.

Another explanation for the association between age and BCR could be normal tissue sparing in younger patients. A recent study observed that larger delineations at the prostate-bladder and prostate-seminal vesicle interfaces, and also the posterior and apex regions of the prostate reduced BCR in patients treated with IMRT (60). The prostate apex was outside of the CTV for all but four brachytherapy patients (**Supplementary Material Figure S7B**), indicating that the association we find between dose and BCR in this region could be a result of contouring uncertainty or bias which should be kept in mind when planning for young patients with aggressive disease treated with brachytherapy boost.

This is the first application of multivariable Cox-IBDM in prostate cancer patients. Not only do we demonstrate the benefit of incorporating dose information into predictive models, we explore the relationship between dose and prognostic variables, and their combined effect on treatment outcome. Our results demonstrate that there is no “one size fits all” for optimum treatment of high-risk prostate cancer patients. We highlight the potential impact of irradiation outside of the CTV via pelvic nodal, seminal vesicle or whole pelvis irradiation, with results suggesting the design of future randomized trials should consider a more personalized approach to treatment decisions, considering different treatment regimens according to patient age for example, rather than limiting themselves to PORT vs WPRT.

Our results are hypothesis generating and should be validated in external datasets. Although our results validate previous observations of an association between extra-prostatic dose and treatment outcome (18, 27), the different anatomical regions identified between studies highlights how dependent upon treatment protocol, center and fractionation IBDM results are. Whether the “fractionation” effect suggested is a true biological effect, or merely a physical effect of different treatment techniques cannot be determined at this point and more work must be done to understand the underlying mechanisms results for IBDM to become generalizable to the wider patient population. It may also be worth noting that our BCR end-point represents a surrogate for treatment response, and does not act as a hard end-point such as metastasis-free or cancer-specific survival, of which was not available to us.

We are confident the identified regions were consistently mapped between patients as the measured registration uncertainty (**Figure S1 Supplementary Material**) was well below the size of the identified region. We also acknowledge that using dose distribution export by the brachytherapy planning system would have been superior to our reconstruction of the brachytherapy dose, however this option was not available. We visually compared sampled of our reconstructed dose distributions with those of plans on the brachytherapy planning system, and observed no fundamental difference in results when randomly shifting the HDR dose distribution by up to 1cm (61) in the SI, AP or LL directions (**Supplementary Material Figure S9**). We therefore deem our dose reconstruction method and associated assumptions to be sufficiently accurate for the purposes of this study.

We further note that we perform analysis using planning CT scans and the planned dose distribution, and do not account for inter- and intra-fractional motion of the prostate and seminal vesicles (47–49). It would be interesting to perform IBDM analysis using inter-treatment images and delivered dose, perhaps taking advantage of the imaging facilities available during magnetic resonance guided radiotherapy (62). However, prostate motion (2-4 mm SD) is small compared with the inter-patient registration uncertainty and unlikely to affect the results, especially because the motion is random and therefore does not bias the result.

We acknowledge our results may have been affected by some patients being excluded from Cox-IBDM due to missing information. However, Cox-IBDM results reflected that of binary-IBDM where no patients were excluded. Finally, although we include commonly identified prognostic variables in this work, there may have been unidentified confounding variables, such as hypoxia score, that may affect results under the **Table 2** fallacy where coefficients in multiple regression models are mutually adjusted and can be misinterpreted (63). To advance predictive modelling using voxel-based analysis and to further explore dose-fractionation relationships, we should consider not only interaction between dose and prognostic variables, but also an individual’s biology.

## 5 Conclusion

IBDM identified regions outside of the target volume where incidental excess dose was significantly associated with reduced BCR for high-risk prostate cancer patients treated with EBRT plus brachytherapy boost. The effect depended on fractionation schedule; no association was detected for patients treated with hypo-fractionated radiotherapy of IMRT.

The first application of multivariable Cox-IBDM for prostate cancer patients revealed that dose response is influenced by prognostic variables. Our results, which are hypothesis generating and should be verified in an external cohort, suggest that we may be undertreating the seminal vesicle tips in patients treated with conformal brachytherapy boost, particularly younger patients. Finally, we demonstrate that risk-stratification models perform significantly better with the addition of dose information.

## Data availability statement

The data analyzed in this study is subject to the following licenses/restrictions: This study was based on data accessed via institutional approval (research ethics committee reference: 17/NW/0060). The authors do not own these data and hence are not permitted to share them in the original form (only in aggregate form, e.g., publications). Requests to access these datasets should be directed to gareth.price@manchester.ac.uk.

## Author contributions

JS: implemented primary analysis and wrote manuscript, data collection. EVO: methodological discussions and reviewed the manuscript, input to image registration. AG: methodological discussions and reviewed the manuscript, developed computational toolkit for IBDM calculations. AW: methodological discussions and reviewed the manuscript. TE: reviewed the manuscript and offered clinical advice, data collection. KR: reviewed the manuscript. CJ-H: performed image registration and initial calculations. WB: performed image registration and initial calculations. PH: reviewed the manuscript and offered clinical advice. AC: reviewed the manuscript and offered clinical advice MH: conceived the experiment, methodological discussions and reviewed the manuscript. All authors contributed to the article and approved the submitted version.
