# Supplementary material for: Dose outside of the prostate is associated with improved outcomes for high-risk prostate cancer patients treated with brachytherapy boost
Source: Front Oncol. 2023 Jun 15;13:1200676. doi: 10.3389/fonc.2023.1200676 (PMC10311256; doi:10.3389/fonc.2023.1200676)
Supplement: Supplementary file 1 [file DataSheet_1.pdf]

	Standard Deviation (cm)		
	LR	AP	SI
Left SV	0.48	0.58	0.90
Right SV	0.49	0.54	0.91
Apex	0.16	0.65	0.63

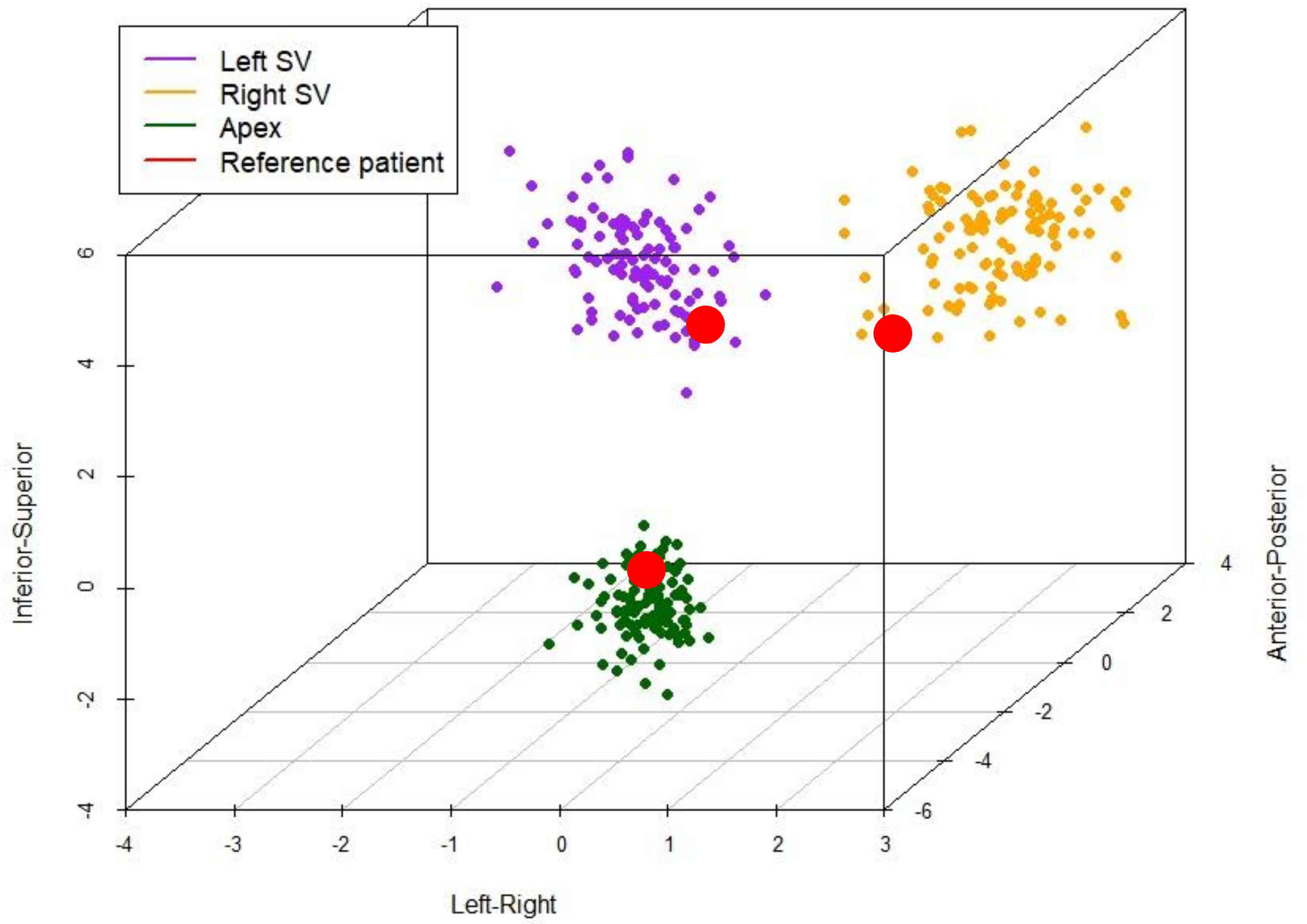

Figure S1: Manual landmarks made on the seminal vesicle tips and inferior region of the prostate for every brachytherapy patient before, shown after dose mapping. The red points indicate marks of the reference patient used for image registration and dose mapping. Dose distributions were blurred according to the standard deviation of the markers in all three dimensions (shown in the table on the left).

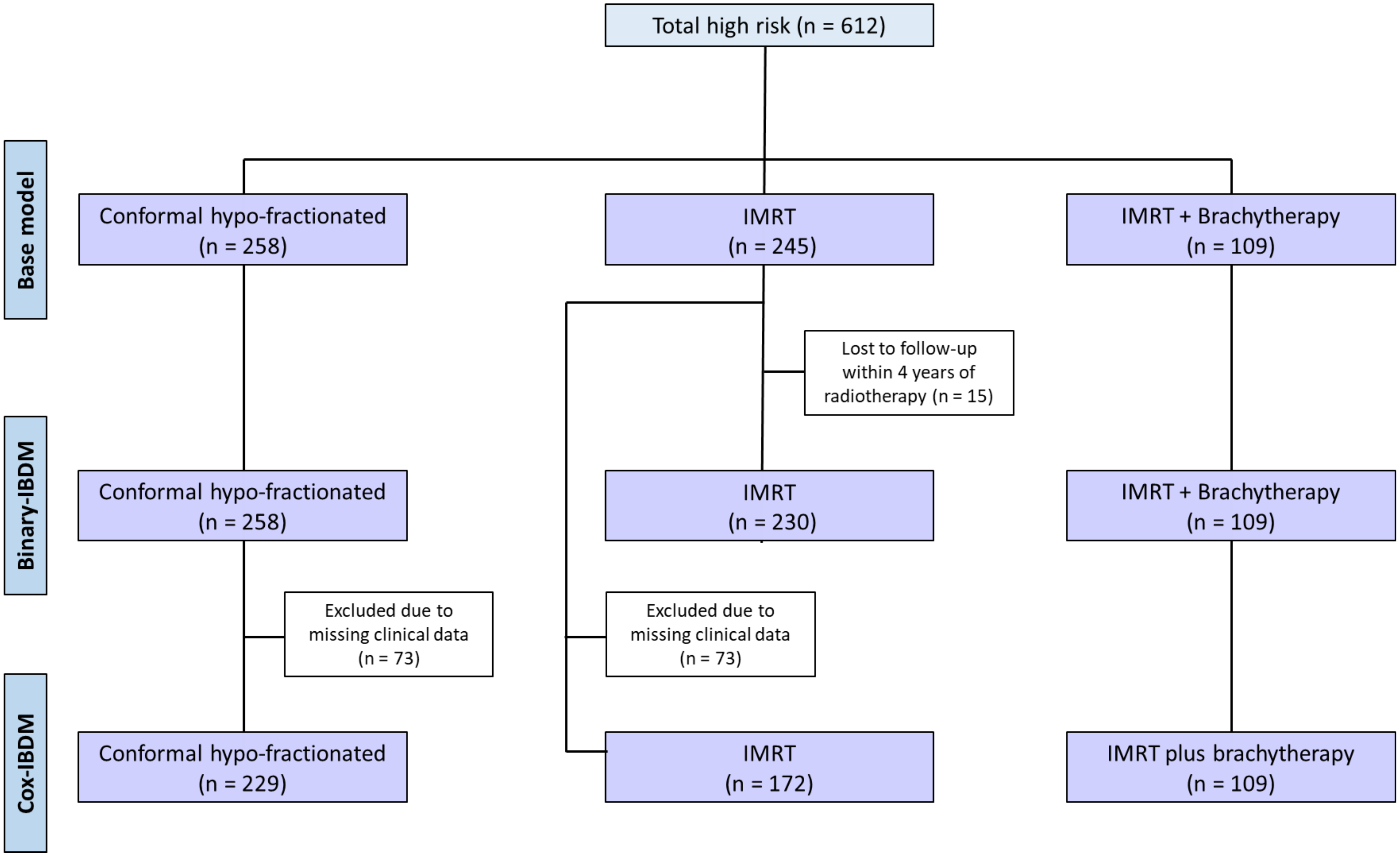

Figure S2: Consort diagram of patients included in each stage of analysis. Patients lost to follow-up within four years of radiotherapy were excluded from binary-IBDM, but not from Cox-IDBM, as this method considers time-to-event. Patients with any number of missing clinical variables were excluded from Cox-IBDM.

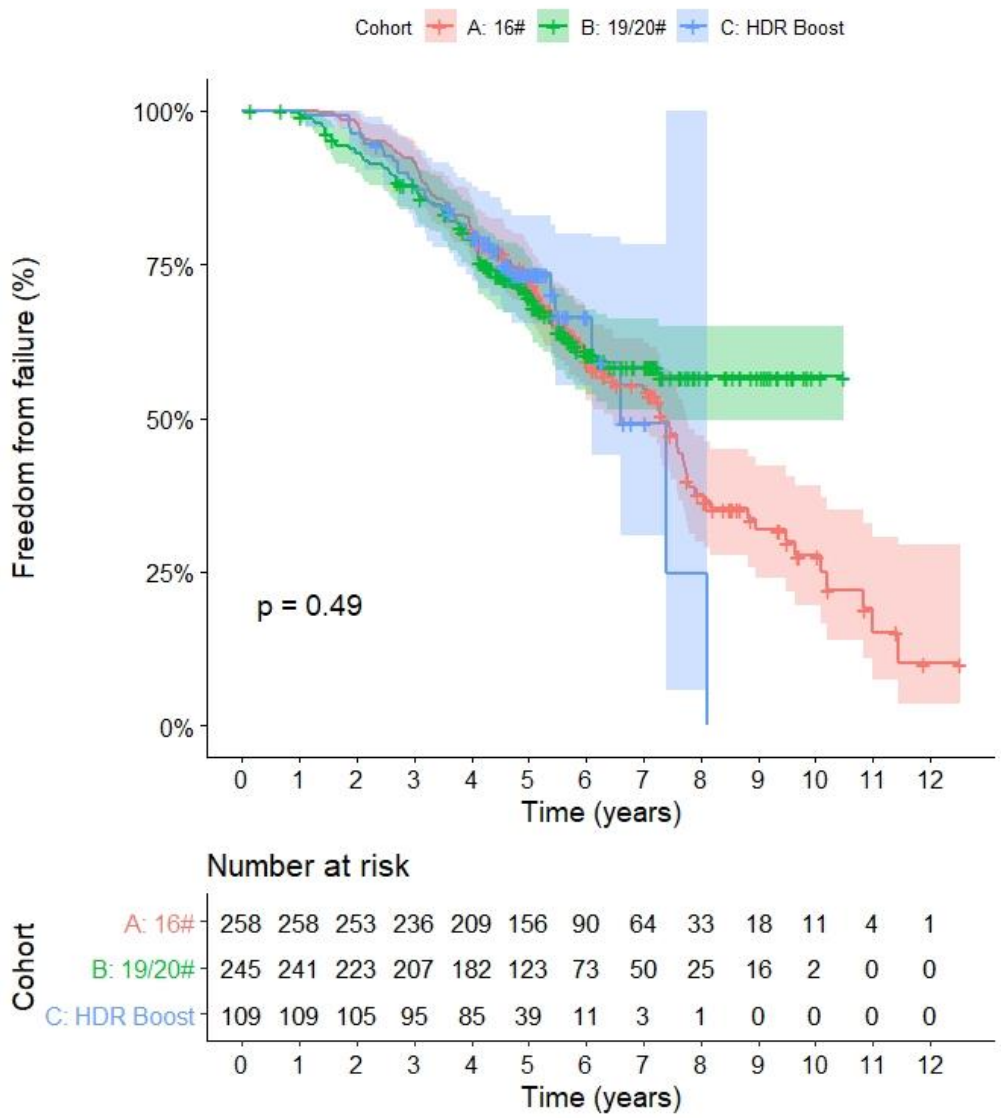

Figure S3: Kaplan-Meier survival curves showing Biochemical recurrence (BCR), stratified by fractionation schedule. There was no significant difference in BCR between the three Cohorts.

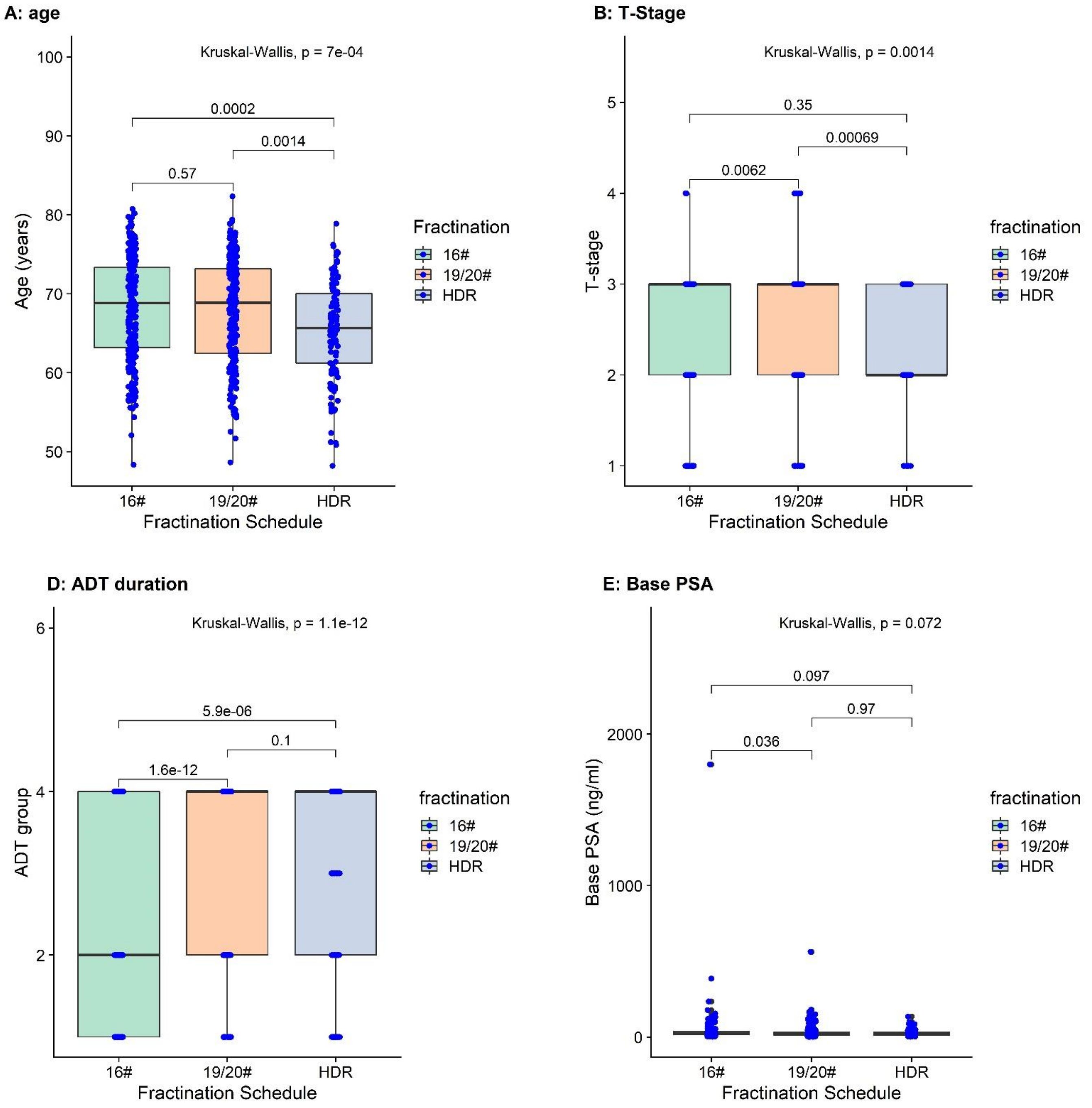

Figure S4: Box-plots showing the distribution of prognostic variables in each fractionation cohort. The distribution of all prognostic variables excluding baseline PSA was significantly different demographics was significantly different from at least one other cohort for patients treated with High dose Rate (HDR) brachytherapy (Wilcoxon test).

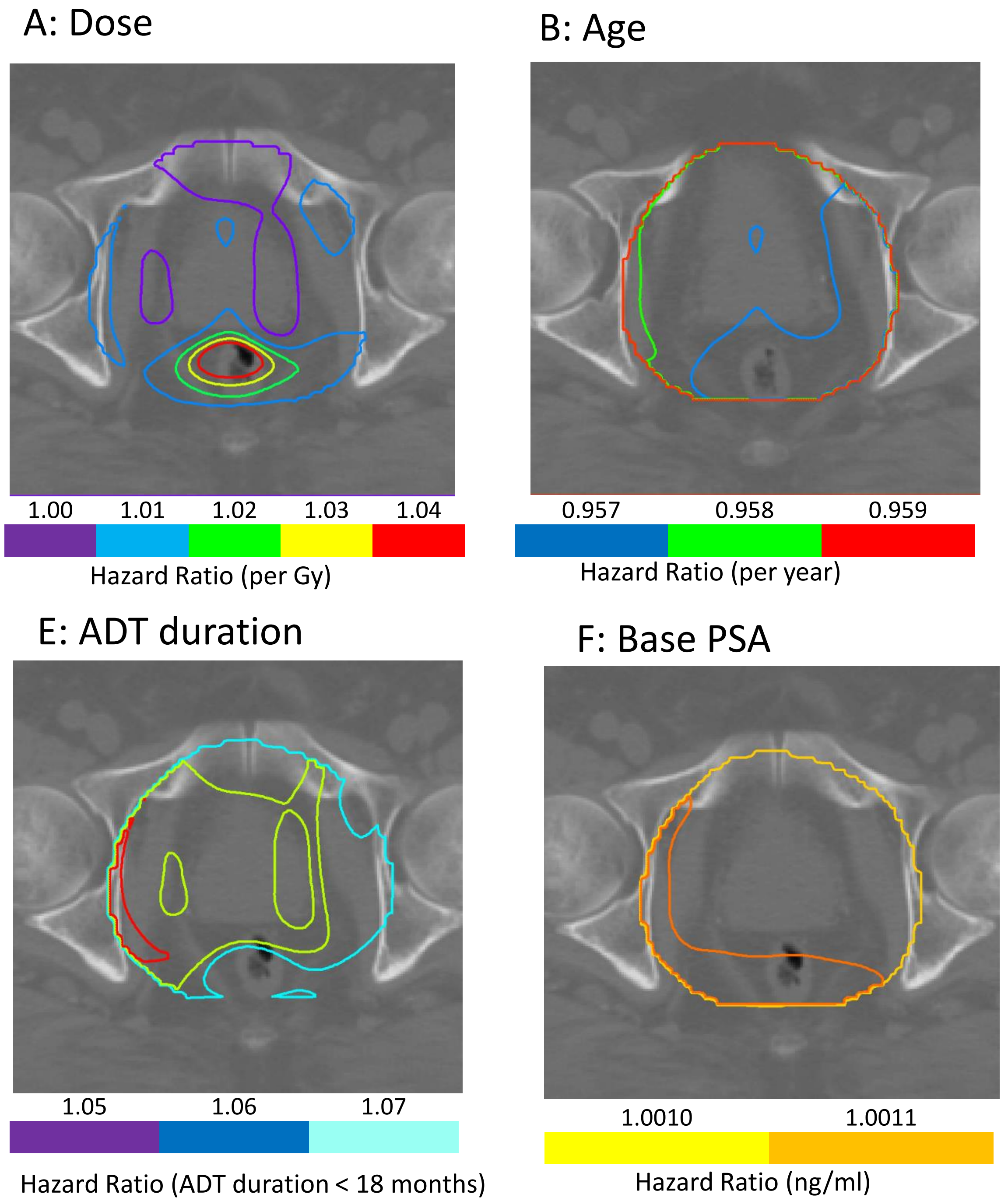

Figure S5: Cox-IBDM calculated HR maps for patients treated with conformal hypo-fractionated radiotherapy (50 Gy in 16 fractions). No voxels were significantly associated with biochemical recurrence for dose or any prognostic variable.

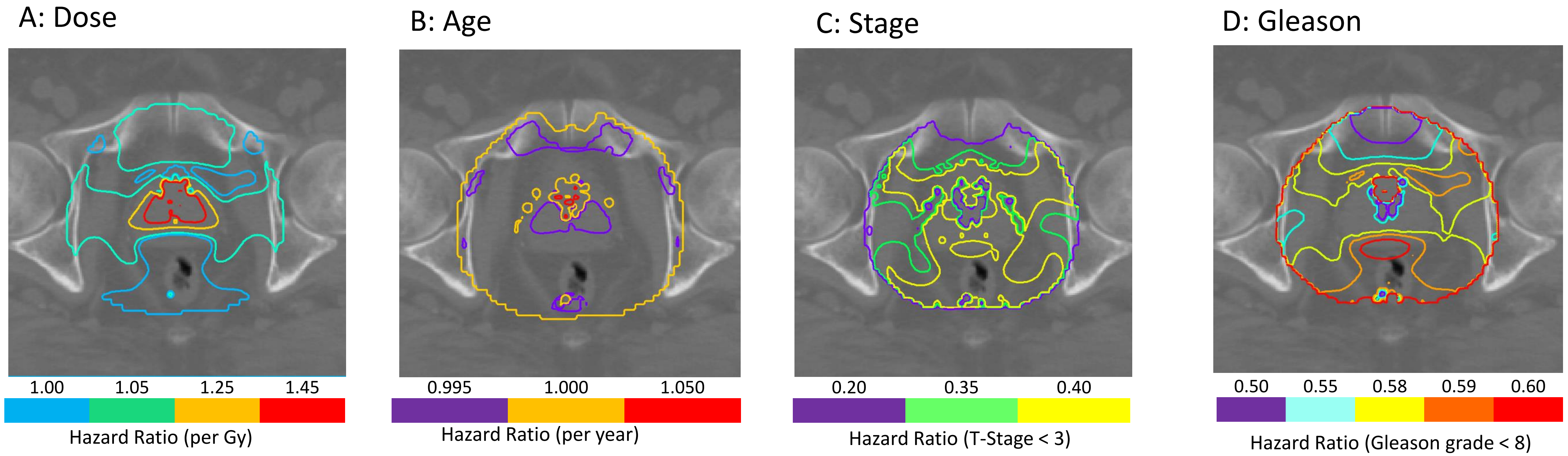

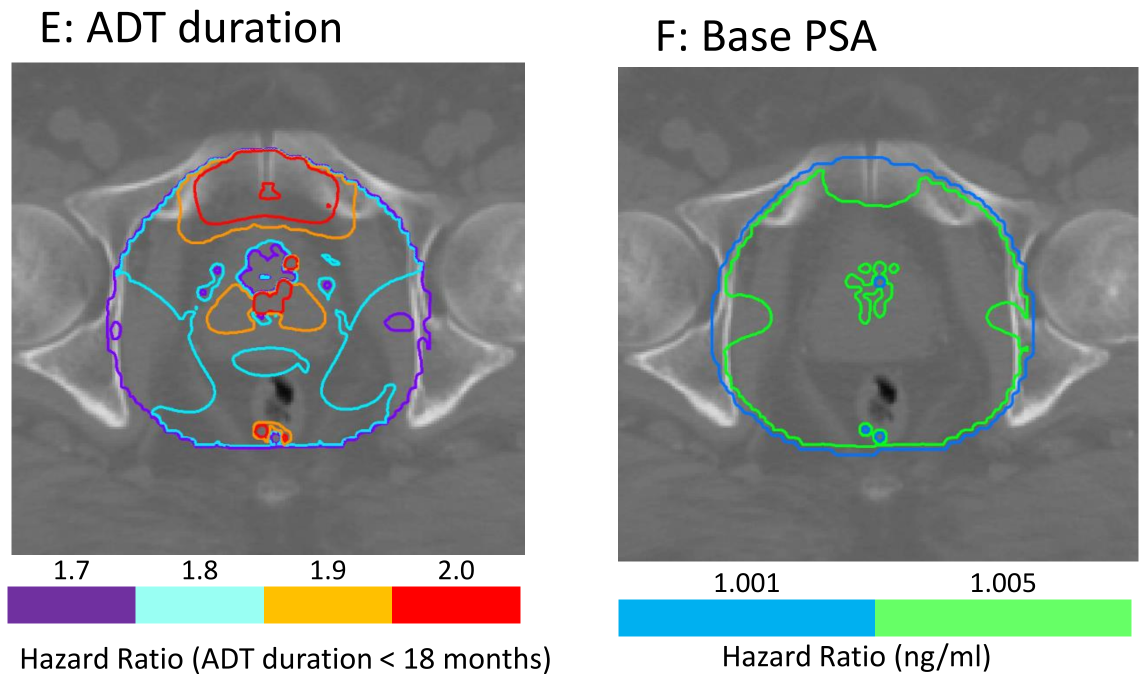

Figure S6: Cox-IBDM calculated HR maps for patients treated with Intensity Modulated Radiotherapy (57/60 Gy in 19/20 fractions). No voxels were significantly associated with biochemical recurrence for dose or any prognostic variable.

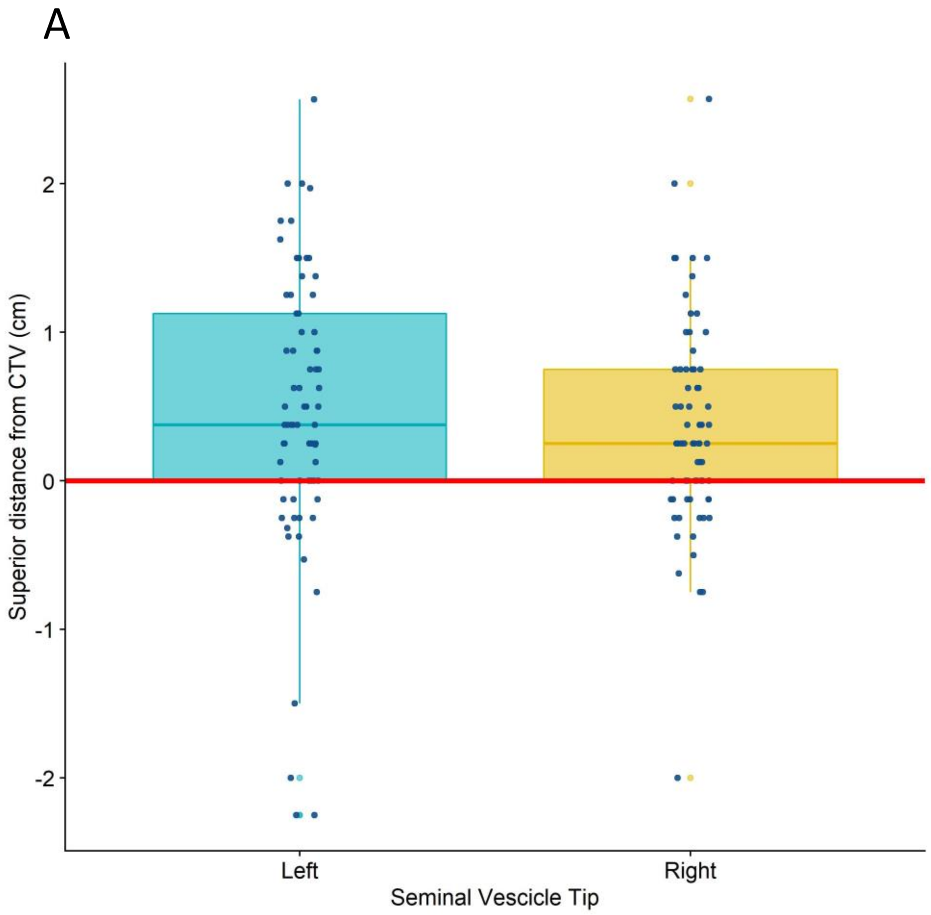

	Distance from CTV (cm)	
	Left SV	Right SV
Mean	0.52	0.39
SD	0.92	0.68

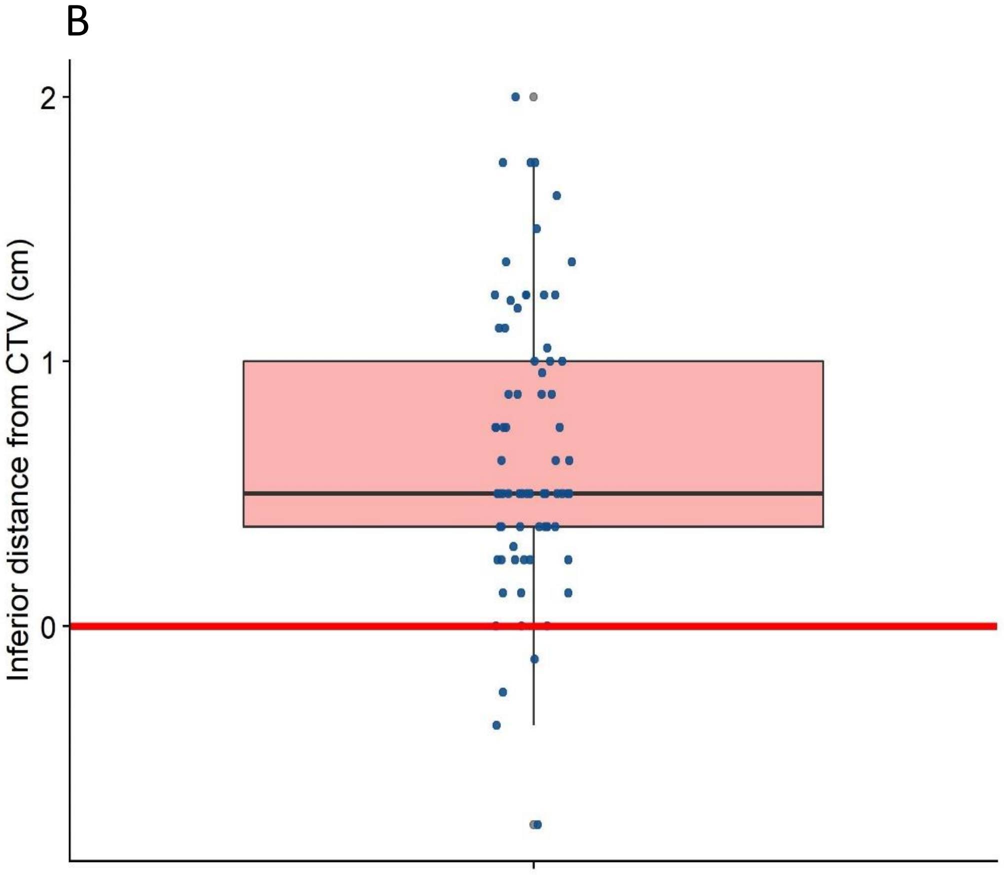

	Distance from CTV (cm)
Mean	0.67
SD	0.53

Figure S7: Boxplot showing the distance between the manually made landmarks at A the seminal vesicle tips (see Figure S1) and B the apex of the prostate and the most superior aspect of the CTV, which was used for dose mapping. The seminal vesicle tips were outside of the CTV for all but 14 patients, and the apex was outside for all but four patients.

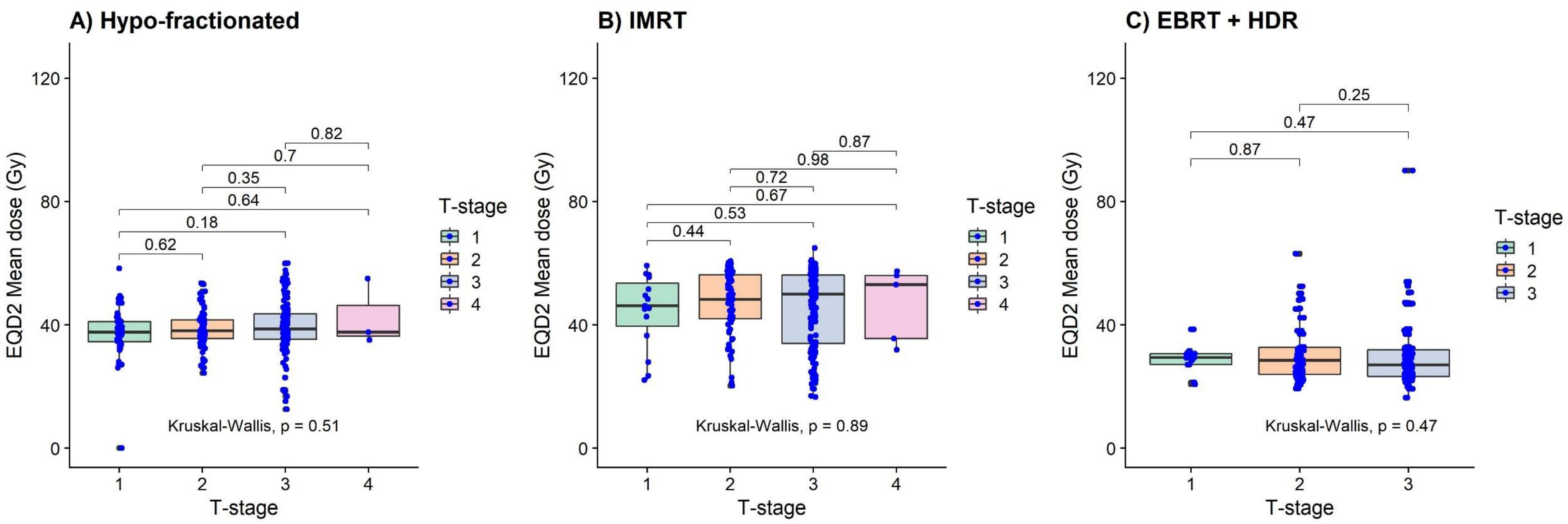

Figure S8: Boxplots showing the distribution of T-stage and mean dose (EQD2) in the "common" region identified as significantly associated with biochemical recurrence in both binary – and Cox-IBDM for patients treated with IMRT plus a single brachytherapy boost, found for all patients. There was no significant difference in mean dose across any T-stage within all three fractionation cohorts.

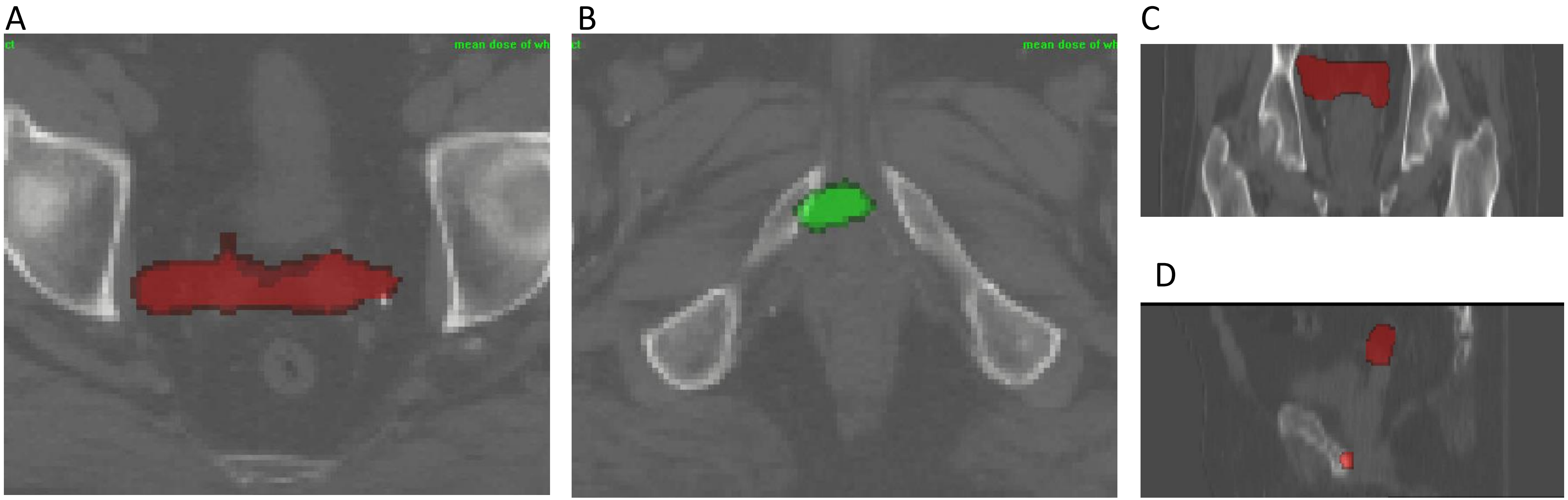

Figure S9: IBDM results (A-D: Binary-IBDM, E-G: Cox-IBDM) for patients treated with IMRT plus brachytherapy boost, with the HDR dose distribution shifted by up to 1cm in any random direction prior to dose mapping. Panels A and B show axial views, with the red and green overlay indicating regions where dose was significantly associated with BCR across the seminal vesicles (A) and apex (B) (p≤0.02). Panels C and D show coronal and sagittal views of the Binary-IBDM regions respectively.

Results are not affected by this exercise.

Table 1S: Multivariable Cox proportional-hazards analysis for clinical prognostic covariates included in the study (age, T-stage, Gleason grade, ADT duration, baseline PSA), dichotomising on ISUP Class ≥3 vs ≤2, i.e.. ≥4+3 vs ≤3+4, as opposed to ≥8 vs <8, i.e. ISUP Class 4-5 vs ≤3. Results presented in the manuscript were not affected by this different Gleason dichotomisation. Analysis was conducted on each fractionation cohort separately.

**Multivariable**	**Conformal hypo-fractionated (50Gy in 16#)**		**IMRT (57/60Gy in 19/20#)**		**EBRT + Brachytherapy (37.5Gy in 15# + 15Gy HDR boost)**	
	**HR (95 % CI)**	**p-value**	**HR (95 % CI)**	**p-value**	**HR (95 % CI)**	**p-value**
Age (continuous)	0.98 (0.95 - 1.01)	0.200	1.00 (0.95 - 1.04)	0.900	0.95 (0.90 - 1.01)	0.090
T-stage (≥T3 reference)	-	-	-	-	-	-
<T3	0.71 (0.48 - 1.05)	0.085	0.44 (0.23 - 0.84)	**0.013**	0.50 (0.24 – 1.04)	**0.063**
Gleason group (≥3 as reference)	-	-	-	-	-	-
<3	0.92 (0.61 - 1.37)	0.700	0.95 (0.52 - 1.74)	0.900	0.48 (0.72 – 3.04)	0.300
ADT duration group (≥18 months as reference)	-	-	-	-	-	-
<18 months	1.14 (0.74 - 1.76)	0.500	1.43 (0.80 – 2.57)	0.200	1.84 (0.85 – 3.98)	0.120
Baseline PSA (ng/ml)	1.00 (1.00 - 1.00)	**0.014**	1.01 (1.00 - 1.01)	**<0.001**	1.01 (1.00 - 1.02)	0.200

HR = Hazard Ratio   CI = Confidence Interval   AIC = 265.3, c-index = 0.664
