# Supplementary material for: Dose outside of the prostate is associated with improved outcomes for high-risk prostate cancer patients treated with brachytherapy boost
Source: Front Oncol. 2023 Jun 15;13:1200676. doi: 10.3389/fonc.2023.1200676 (PMC10311256; doi:10.3389/fonc.2023.1200676)
Supplement: Supplementary file 2 [file DataSheet_2.docx]

Supplementary Material

Dose outside of the prostate is associated with improved outcomes for high-risk prostate cancer patients

**Jane Shortall^1^, Eliana Vasquez Osorio^1^, Andrew Green^1^, Alan McWilliam^1,2^, Thriaviyam Elumalai^2^, Kimberley Reeves^1^, Corinne Johnson-Hart^2^, William Beasley^2^, Peter Hoskin^2,1^, Ananya Choudhury^1,2^ and Marcel van Herk^1^**

^1^The University of Manchester Department of Cancer Sciences, Manchester UK

^2^The Christie NHS Foundation Trust, Manchester UK

*** Correspondence:**Jane Shortall
jane.shortall@manchester.ac.uk

# Appendix A: Detailed Method

1. **Study Design**

A total of 612 patients with high-risk prostate cancer (Gleason grade ≥8, tumour stage (T-stage) ≥T3a or baseline prostate-specific antigen (PSA) ≥20ng/ml^1^) treated with radiotherapy between 2005 and 2013 at a single academic centre were included in this study. Patients were treated with either conformal hypo-fractionated radiotherapy (50 Gy in 16 fractions, n=258, prostate and seminal vesicles irradiated), hypo-fractionated Intensity Modulated Radiotherapy (IMRT) (57Gy or 60Gy in 19 or 20 fractions respectively, n=245, prostate and seminal vesicles irradiated inline with CHHiP guidelines ^2^), or IMRT (37.5 Gy to the prostate in 15 fractions) plus a 15 Gy single fraction High Dose Rate (HDR) brachytherapy boost to the prostate (n=109). Treatment fractionation schedule was assigned by a consultant clinical oncologist according to local practice.

The planning Computed Tomography (CT) scan and delineations, 3D planned dose distribution and patient and tumour characteristics (age, T-stage, Gleason grade, Androgen Deprivation Therapy (ADT) duration and baseline PSA) were collected for all patients. Radiotherapy planning data was collected from the Philips Pinnacle treatment planning system archive. Institutional approval had been granted to use this data (research ethics committee reference: 17/NW/0060).

The end-point of the study was BCR, defined according to the American Society for Therapeutic Radiology and Oncology (ASTRO) recommendations (PSA nadir + 2ng/ml)^3^. To investigate the effect of fractionation on the dose BCR relationship and avoid spurious results caused by the inherently different radiotherapy techniques, the three cohorts were analysed separately.

- 1. **Brachytherapy dose reconstruction**

For those treated with IMRT plus brachytherapy, the brachytherapy boost was planned using ultrasound imaging and a single PTV to the prostate, and delivered with a HDR treatment using an Ir-192 source. As the dose distribution export were not supported by the brachytherapy planning system, the planned brachytherapy dwell positions and catheter positions and times were collected and used to reconstruct the planned dose. The coordinates and time for each source were used to calculate the dose around each position using a simple inverse square law function. Weighted dwell times at time of treatment were calculated using a nominal 10 Ci Ir-192 source and conversion factors according to IPEM code of practice^4^. As to replicate clinical procedure, the AAPM TG43 line source model and parallel needles were assumed^5^. The dose distributions of each dwell position were then summed, accounting for contributions from all sources, to provide the total dose.

1. **Dose Mapping**

For patients treated with brachytherapy boost, the reconstructed dose distribution was spatially aligned to the external beam planned dose distribution by aligning the centre of gravity of the dwell positions with the centre of gravity of the prostate in the planning CT. Note that the centre of gravity of the prostate volume and HDR dose distributions were aligned assuming that the HDR dose was prescribed to the prostate volume, with the highest dose region at the centre of the prostate.

The total treatment dose was then summed using equivalent dose in 2-Gy fractions (EQD2) (alpha-beta ratio=1.5 Gy)^6^. Note that, as analysis explored relative differences between dose distributions of the same treatment technique and not absolute dose, the alpha-beta ratio used acts merely as a scaling factor with no impact on our results^7^. Alignment was visually spot checked for 10 patients to ensure the 15 Gy line aligned with the prostate contour. Note that, as our analysis explored relative differences between dose distributions of the same treatment technique and not absolute dose, the alpha-beta ratio used acts merely as a scaling factor with no impact on our results.

All planning CTs were then spatially registered to an arbitrarily chosen reference patient using the same algorithm as Witte *et al*.^8^ (in-house software). The algorithm mapped the Clinical Target Volume (CTV) delineation of each patient to that of the reference patient, where all voxels were scaled radially between the centre of gravity of the reference CTV and the respective prostate contours. All voxels outside the CTV were mapped radially, maintaining distances (no scaling). This mapping was then used to transform the corresponding dose distribution to the same anatomy. A region of interest forming a sphere of approximately 10cm radius, centred on the centre of the reference prostate, was chosen for analysis to remove regions where spurious results could occur due to differences in radiotherapy plans depending on patient anatomy.

Prior to dose mapping, dose distributions of all patients were flipped in the left-right direction and included in analysis twice. This method, which has been commonly used in other IBDM studies^9,10^, assumes that the likelihood of microscopic disease is symmetric, avoids spurious laterality biased results caused by small asymmetries in the dose distributions, and improves statistical power.

To assess and account for the accuracy of the dose mapping, Target Registration Error analysis was performed ^11^. The left and right seminal vesicle tips and the apex of the prostate were manually indicated on the planning CT of all patients treated with brachytherapy (in-house software). The anatomical accuracy of the landmarks was visually inspected by a clinical oncologist. The uncertainty of the dose mapping was estimated using the standard deviation of the landmarks in the left-right (LR), anterior-posterior (AP), and superior-inferior (SI) directions (supplementary material Figure S1). These standard deviations were used to blur the mapped dose prior to the IBDM by convolving each dose distribution with an anisotropic Gaussian filter. Note that, as the same dose mapping algorithm was used for all three cohorts, the registration uncertainty was assumed to be consistent across all patients.

1. **Voxel-Based Analysis**

Binary-IBDM and Cox-IBDM were performed to assess differences in the dose distributions of patients who did and did-not recur using an in-house developed toolkit^12^. We performed both binary- and Cox-IBDM in order to first validate Witte *et al*’s. results, and to simultaneously include clinical variables in the analysis.

For this, mapped doses were grouped depending on the patient’s BCR status (PSA failure free survival (bNED)=0, fail=1). IBDM was performed for each fractionation cohort. To correct for multiple testing^12–14^ , whereby simultaneously testing many variables (each voxel) against a single outcome (BCR) can result in inflation of type 1 error meaning some variables appearing significant by chance, and to determine regions of significance, permutation testing was performed^12,14,15^.

Briefly, event labels were permuted relative to dose in each voxel, and a distribution of summary statistics (T or β statistics) for the entire dose distribution constructed to represent the null hypothesis that there is no difference between the two groups. A threshold on the summary statistic was then used to test for significant association in each voxel of the unpermuted data. We direct the reader to ^12,14–16^ for more detailed explanation of the statistical methods used.

**4.1 Binary-IBDM**

As binary-IBDM does not consider time-to event, we chose a cut-off point after radiotherapy of four-years, consistent with Witte *et al.*^8^. Mapped doses for each voxel of the bNED and fail groups were compared using a Students T-test. T-maps containing the observed t-values in each voxel were created. A negative t-value indicates that excess dose is associated to BCR. Conversely, a positive t-value indicates that excess dose is associated to lack of BCR.

In this setting, permutation testing implied randomly permuting event labels 1,000 times and repeating the process to create 1,000 null hypothesis t-maps. Each permutation was summarised using the most extreme positive and negative t-statistic (extreme voxel values in the t-map). The ranking of a given t-statistic among the permuted t-statistics maps gives a p-value. Iso-t-levels indicating significance were then plotted on the observed t-map to identify regions where the dose distributions of patients who did and did not recur significantly differed^12,15^.

**4.2 Cox-IBDM**

To account for the time to BCR and other prognostic clinical variables when identifying regions where the dose distributions of patients who did and did-not fail significantly differed, Cox-IBDM was performed. For this, dose distributions were categorised as fail and bNED based on recurrence at any point during follow-up. Note that 15 patients treated with IMRT were censored at this stage due to being lost to follow-up. A Cox proportional-hazards model was constructed for each voxel, including mapped doses to that voxel, age (continuous), T-stage (≥ T3 vs < T3), Gleason grade (≥ 8 vs < 8), ADT duration (≥ 18 months vs < 18months), and baseline PSA (continuous).

Similar to binary-IBDM, the resulting β-statistics were collected to create β-maps, and permutation testing, where event labels and time to recurrence were permuted against clinical variables, was performed, collecting the extreme β -statistic for each included variable. Iso-β levels indicating significance were plotted on the observed β-map for each variable (see Green *et al*. for more detail ^12^). To ease interpretability, the observed β-maps and was transformed to Hazard Ratio (HR) maps, by applying the exponential.

Patients with one or more missing prognostic variables were excluded from Cox-IBDM analysis. The consort diagram in Figure S2 supplementary material summarises patients included in each stage of the analysis. Note that patients lost to follow-up within four years of radiotherapy were excluded from binary-IBDM but included in Cox-IBDM.

1. **Statistical Analysis**

Baseline univariable and multivariable Cox proportional-hazard models including prognostic variables only were created (age, T-stage (≥T3 vs <T3), Gleason grade (≥8 vs <8), ADT duration (≥18 months vs <18 months), and baseline PSA). To investigate the impact of including dose information in our predictive models, mean dose in regions identified as significantly associated with BCR was included in univariable and multivariable Cox proportional-hazard models. To avoid our model becoming over-complicated, we chose to only include mean dose in those regions commonly identified as significantly associated with BCR for both binary- and Cox-IBDM. With different end-points (binary vs time-to-event), and Cox-IBDM including cofounders, the overlap of the binary-IBDM regions with the better-defined Cox-IBDM regions was considered to provide confidence of a true result.

The Akaike information criterion (AIC) and concordance-index (c-index) were calculated to compare the performance of models that did and did-not include dose information.

All statistical analysis was performed using R (version 4.0.2) in RStudio (desktop version 1.3.1073)

1. Ekanger, C. *et al.* Ten-Year Results From a Phase II Study on Image Guided, Intensity Modulated Radiation Therapy With Simultaneous Integrated Boost in High-Risk Prostate Cancer. *Adv. Radiat. Oncol.* **5**, 396–403 (2020).

2. Dearnaley, D. *et al.* Conventional versus hypofractionated high-dose intensity-modulated radiotherapy for prostate cancer: 5-year outcomes of the randomised, non-inferiority, phase 3 CHHiP trial. *Lancet Oncol.* **17**, 1047–1060 (2016).

3. Roach, M. *et al.* Defining biochemical failure following radiotherapy with or without hormonal therapy in men with clinically localized prostate cancer: Recommendations of the RTOG-ASTRO Phoenix Consensus Conference. *Int. J. Radiat. Oncol. Biol. Phys.* **65**, 965–974 (2006).

4. Bidmead, A. M. *et al.* The IPEM code of practice for determination of the reference air kerma rate for HDR 192Ir brachytherapy sources based on the NPL air kerma standard. *Phys. Med. Biol.* **55**, 3145 (2010).

5. Rivard, M. J. *et al.* Update of AAPM Task Group No. 43 Report: A revised AAPM protocol for brachytherapy dose calculations. *Med. Phys.* **31**, (2004).

6. van Leeuwen, C. M. *et al.* The alfa and beta of tumours: A review of parameters of the linear-quadratic model, derived from clinical radiotherapy studies. *Radiation Oncology* vol. 13 (2018).

7. Vásquez Osorio, E. M. *et al.* Three-dimensional dose addition of external beam radiotherapy and brachytherapy for oropharyngeal patients using nonrigid registration. *Int. J. Radiat. Oncol. Biol. Phys.* **80**, 1268–1277 (2011).

8. Witte, M. G. *et al.* Relating dose outside the prostate with freedom from failure in the Dutch trial 68 Gy vs. 78 Gy. *Radiat. Oncol. Biol.* **77**, 131–138 (2010).

9. Beasley, W. *et al.* Image-based Data Mining to Probe Dosimetric Correlates of Radiation-induced Trismus. *Int. J. Radiat. Oncol. Biol. Phys.* **102**, 1330–1338 (2018).

10. Jenkins, A. *et al.* Novel Methodology to assess the Effect of Contouring Variation on Treatment Outcome. *Med. Phys.* **48**, mp.14865 (2021).

11. Brock, K. K., Mutic, S., McNutt, T. R., Li, H. & Kessler, M. L. Use of Image Registration and Fusion Algorithms and Techniques in Radiotherapy: Report of the AAPM Radiation Therapy Committee Task Group No. 132. *Med. Phys.* (2017) doi:10.1002/mp.12256.

12. Green, A., Vasquez Osorio, E., Aznar, M. C., McWilliam, A. & van Herk, M. Image Based Data Mining Using Per-voxel Cox Regression. *Front. Oncol.* **10**, 1178 (2020).

13. Chen, C., Witte, M., Heemsbergen, W. & Herk, M. V. Multiple comparisons permutation test for image based data mining in radiotherapy. *Radiat. Oncol.* **8**, 293 (2013).

14. Jane Shortall, Giuseppe Palma, Hitesh Mistry, Eliana Vasquez Osorio, Alan McWilliam, Ananya Choudhury, Marianne Aznar, Marcel van Herk, A. G. Flogging a Dead Salmon? Reduced Dose Posterior to Prostate Correlates With Increased PSA Progression in Voxel-Based Analysis of 3 Randomized Phase 3 Trials-Marcello et al. *Int. J. Radiat. Oncol. Biol. Phys.* **110**, 696–699 (2021).

15. Palma, G., Monti, S. & Cella, L. Voxel-based analysis in radiation oncology: A methodological cookbook. *Phys. Medica* **69**, 192–204 (2020).

16. Groppe, D. M., Urbach, T. P. & Kutas, M. Mass univariate analysis of event-related brain potentials/fields I: A critical tutorial review. *Psychophysiology* **48**, 1711–1725 (2011).
